# A Simplified and Practical Surgical Treatment for Medial Ectropion: A Case Report

**DOI:** 10.1097/GOX.0000000000002102

**Published:** 2019-05-21

**Authors:** Massimo Pinelli, Marta Starnoni, Giorgio De Santis

**Affiliations:** From the Division of Plastic Surgery, University of Modena and Reggio Emilia, Largo Pozzo 71, 41124 Modena, Italy

## Abstract

We present the case of a 81-year-old patient with right facial palsy suffering from recurrent medial ectropion with lower lateral dislocation of the lacrimal punctum causing epiphora and photophobia. The patient was first treated for ectropion with lateral tarsal strip procedure. Unfortunately, this surgical procedure did not get the expected result. This was the reason we used the Mitek anchor system to fix the lower lateral dislocation of the punctum. We had no recurrence of symptoms during a follow-up period of 18 months.

## INTRODUCTION

Ectropion is one of the most common facial paralysis features. A long-lasting correction of ectropion requires a surgical solution. The surgical purpose is to correct the horizontal lid laxity. Among the lateral canthal tightening techniques, the lateral tarsal strip^1^ remains one of the most commonly used for its simplicity and effectiveness. Nevertheless, it can cause the lateral displacement of the inferior punctum with lacrimal malfunction that requires a surgical correction.

We present a case of a patient treated for ectropion using lateral tarsal strip procedure. Due to this procedure, the patient presented a persistent medial ectropion with lateral dislocation of the lacrimal punctum. The purpose of our surgical procedure was the correction of medial ectropion and the position of the punctum consequentially.

## PATIENT

**Fig. 1.**
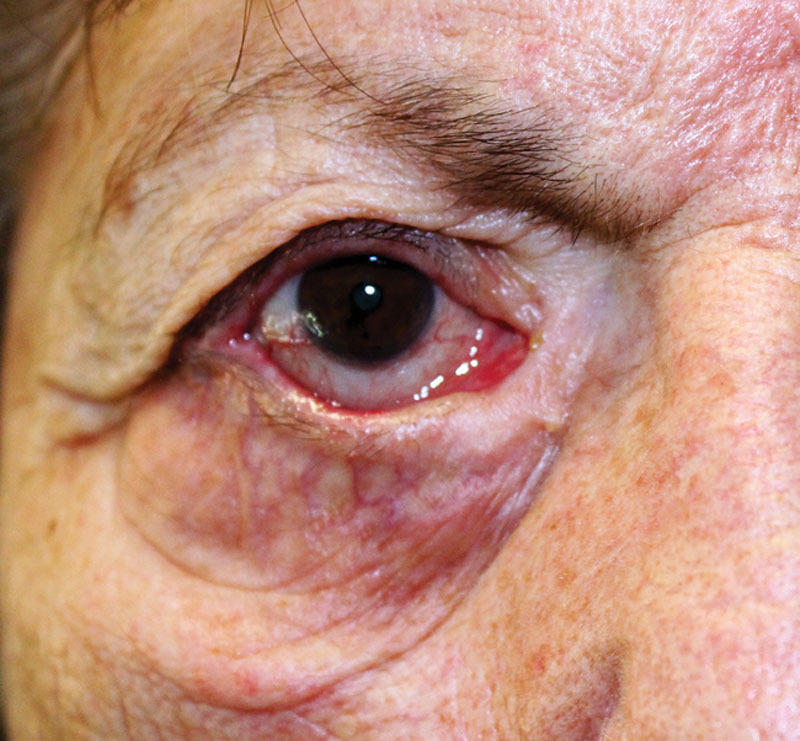
Pre-op view of the patient affected by right medial ectropion.

An 81-year-old patient suffered from a medial ectropion with the punctum outward and laterally displaced (Fig. 1). She referred that she had been suffering from a facial paralysis since she was 2 years old. Nine years before she underwent a lateral tarsal strip procedure. Despite a successful and long-lasting result in the lateral aspect of the lid, medially she got a recurrent ectropion with an outward displacement of the lacrimal punctum. Clinically she complained of epiphora and photophobia. The lacrimal malfunction caused corneal drying and irritation with recurrent conjunctivitis. To fix the anatomical medial canthal position, Mitek anchor system (DePuy Mitek, Raynham, MA) was used, achieving a right lacrimal outflow.

## OPERATIVE TECHNIQUE

The procedure was carried out under general anesthesia.

A 10-mm incision is undertaken from the medial canthal corner to the medial root of the nose without interruption of the medial canthal ligament and the lacrimal pipe.

A little amount of skin of the inferior margin of the incision is removed to expose the tarsal plate. Using a blunt dissection just above the axis of the medial canthal ligament, a small area of the periosteum of the frontal process of the maxilla is exposed, incised, and pilled off.

**Fig. 2.**
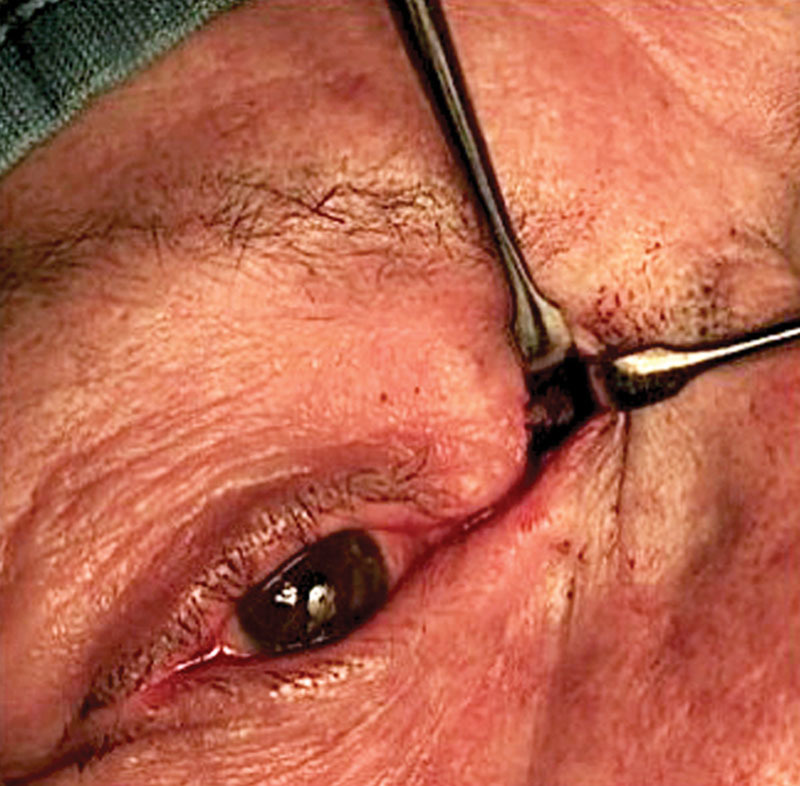
Surgical field showing the bone fixation point.

Using the Mitek Micro Quick Anchor system (DePuy Mitek, Raynham, MA), a hole is drilled 1.5 mm above the medial canthal ligament and the anchor is inserted into the nasal bone (Fig. 2). Putting in traction the connected 4-0 nonabsorbable suture, the bone fixation of the anchor is achieved.

**Fig. 3.**
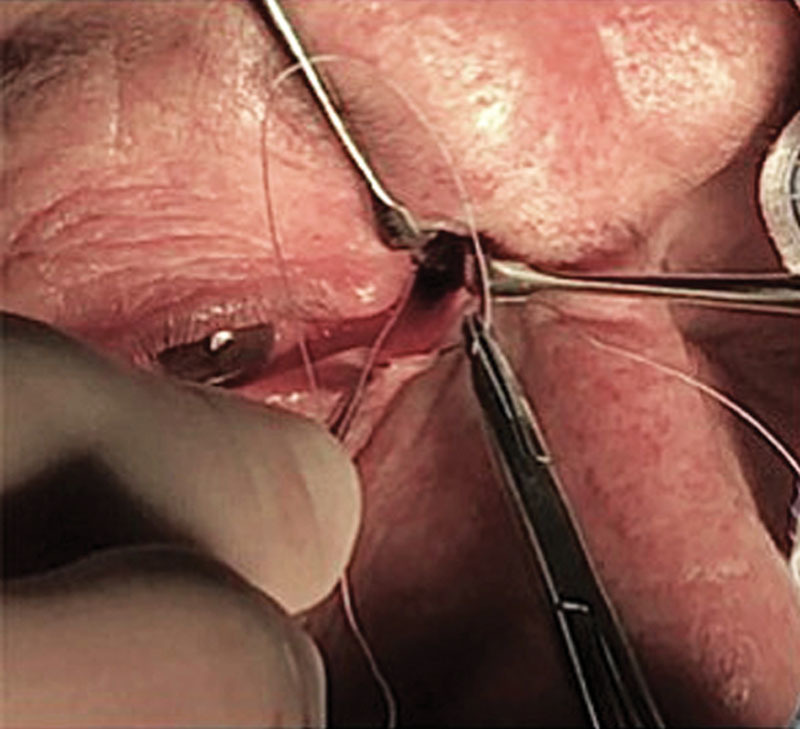
The transfixing tarsal plate suture.

One of the 2 curved needles at both ends of the suture is cut off and the other one is passed through the tarsal plate medially to the punctum (Fig. 3). The knot is tightened till the distance between the punctum and the anchoring point is 10 mm, whereas in the unaffected eye the corresponding distance is 12 mm. During the 6 months after surgery, the achieved result tends to lose efficacy so this overcorrection is necessary. A double layer suture is placed using 5-0 absorbable suture for the orbicular plane and 6-0 interrupted suture for the skin.

## RESULT

**Fig. 4.**
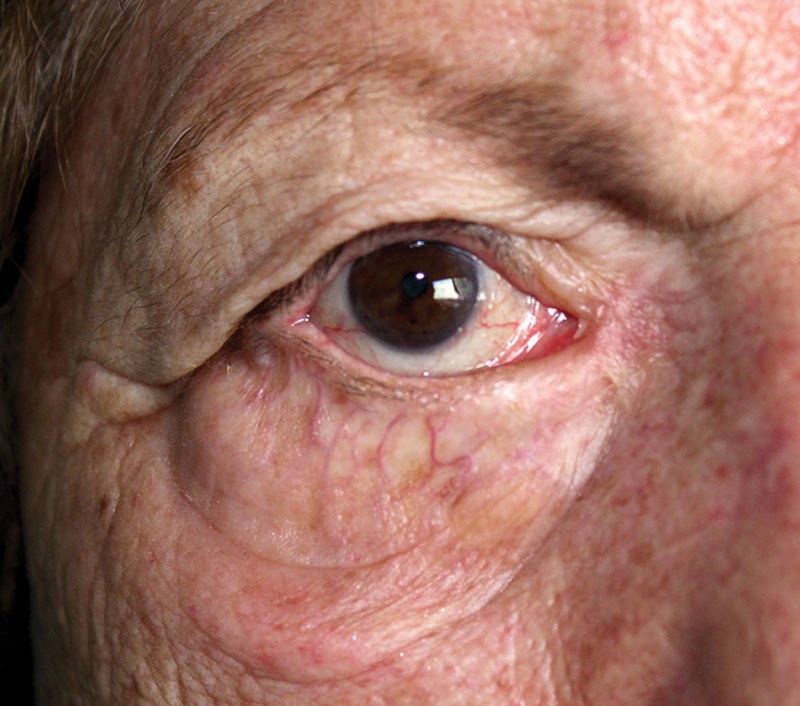
18-month follow-up.

The surgical procedure was completed in 20 minutes. In the postoperative evaluation, an adequate resolution of the ectropion was noticed with the punctum in its normal anatomical position. No recurrence of symptoms occurred during a follow-up period of 18 months. The postoperative period was free of complication related to the anchor. No chronic reaction or extrusion of the suture was noted. The scar did not affect movements of the lid. The patient was satisfied with the result for minimal postoperative assessment and for the disappearance of epiphora and recurrent conjunctivitis (Fig. 4).

## DISCUSSION

Lateral tarsal strip (LTS)^1^ is now frequently performed to treat paralytic ectropion.^2^ This technique has undergone modifications to improve its effectiveness, to reduce complications, and to diminish recurrence rate.^3^

In our patient, the LTS performed 9 years before has solved the lateral ectropion but dislocated laterally the punctum causing epiphora which was the most disturbing symptom. The punctum was dislocated outwardly and this means that LTS was not effective for the treatment of medial ectropion.

Mild degrees of ectropion can be treated by LTS, but in moderate or severe cases a lateral dislocation of the punctum could be caused by the surgical technique itself with possible persistent medial ectropion. To avoid this complication, a surgical correction in the medial eyelid region is advised.

Due to changes affecting the eyelid tissues because of aging and considering the patient’s unwillingness for a long postoperative period, we decided to use the Mitek anchor system. The use of suture anchor for ectropion correction is well described.^4–8^ In particular, suspension of the lower eyelid in facial paralysis using Mitek anchor system has a lot of advantages such as its simplicity, speed, and minimal invasivity.^9^

We used the Mitek anchor system to avoid further damages to the lower eyelid already caused by aging or paralysis and not to short the tarsus that has been reduced by LTS. Because there is no resection of lid tissue and no deformity of lid, in the case of recurrence or insufficient correction of the ectropion other procedures can be performed. The knot is tightened to achieve symmetry between the punctum of the affected and the unaffected lower eyelid so that the lacrimal canaliculus is not obstructed. The bone fixation guarantees a long-lasting result with minimal implanted materials and minimal risk of extrusion. This technique does not include harvest of fascia or other autologous tissues. The postoperative period is short and no specific care is requested. This technique due to its simplicity could be performed simultaneously with LTS for severe degrees of ectropion.

## CONCLUSIONs

LTS is a commonly used procedure, but this technique does not resolve the medial ectropion. Our procedure could be performed in addition to LTS due to its good result even if it was used in only one case. In fact this technique is able to reposition the medial canthal region without invasive approach, leaving the possibility to undertake an additional procedure if required later. It is practicable, simple, fast, and with a low risk of complications. It is important to be careful not to damage the lacrimal system, which may lead to persistent epiphora.
